# Cutaneous metastasis of hepatocellular carcinoma with novel dermoscopic findings

**DOI:** 10.1016/j.jdcr.2025.03.024

**Published:** 2025-04-11

**Authors:** Vixey Silva, Rosemary Oaks, Heather Kopecky, Richard Miller, Brandon Shutty, Jill Browning

**Affiliations:** a Department of Dermatology, HCA Healthcare/USF Morsani College of Medicine: Largo Medical Center Program, Largo, Florida; b Department of Dermatology, C.W. Bill Young VA Medical Center, Bay Pines, Florida

**Keywords:** cutaneous metastasis, dermoscopy, hepatocellular carcinoma, rainbow sign

## Clinical presentation

**Fig 1:**
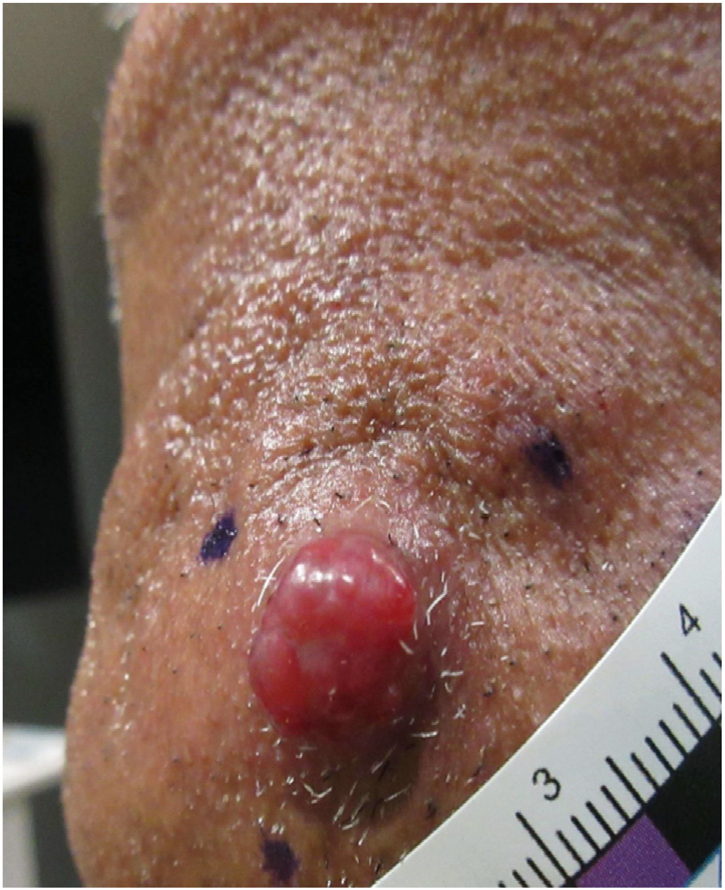
Two centimeter, *red*, shiny nodule on the left chin.

A 66-year-old man with a history of hepatocellular carcinoma (HCC) and hepatitis B presented with a 2 cm, asymptomatic chin nodule growing rapidly for 2 months (Fig 1).

## Dermatoscopic appearance

**Fig 2:**
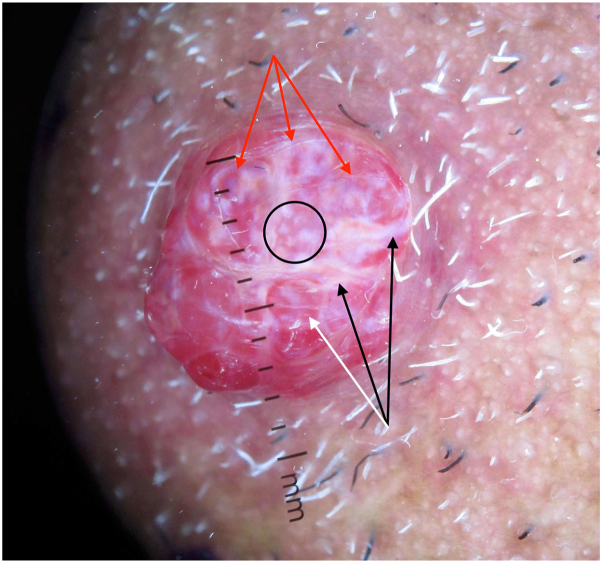
Dermoscopic image under polarized light and direct contact showing a red homogeneous lesion with a white veil, multiple chrysalis-like shiny *white lines* (*black arrows*), *white dots* (*circled*), a positive rainbow sign (*red arrows*), and a surrounding skin-colored collarette. *Red* lacunae are also visualized (*white arrow*).

Dermoscopy showed a diffuse red homogeneous lesion with a white veil, chrysalis-like shiny white lines, white dots, red lacunae, and a skin-colored collarette (Fig 2). A positive rainbow sign was present.

## Histologic diagnosis

**Fig 3:**
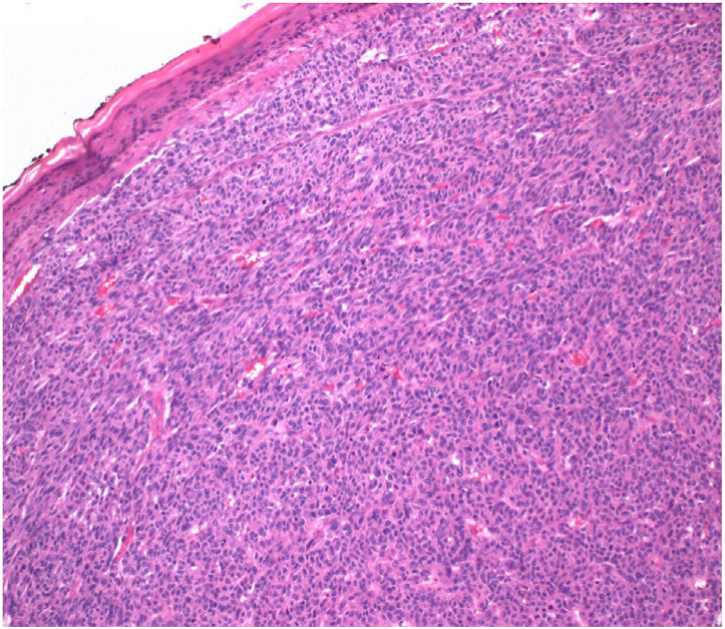
Histopathological image revealing atrophic epidermis with effaced rete ridges, islands of eosinophilic cells with small pleomorphic nuclei, and numerous small vascular spaces.

Histopathology revealed an atrophic epidermis with effaced rete ridges, islands of eosinophilic cells with pleomorphic nuclei, and numerous vascular spaces with extravasated red blood cells (Fig 3).

**Key message**

Cutaneous metastasis of HCC is rare, accounting for less than 0.8% of all cutaneous metastases, but its recognition is crucial for detecting internal malignancy.^1^ These metastases typically present as firm, painless, red or bluish papules or nodules of varying sizes on the face, scalp, chest, or shoulders, sometimes with ulceration. This case highlights novel dermoscopic findings, including crystalline structures and the rainbow sign, previously unreported in HCC metastases. These unique features, along with red lacunae, white dots, and disorganized vessels, can help differentiate cutaneous HCC metastasis from clinical mimickers such as amelanotic melanoma and vascular tumors (eg, lobular capillary hemangioma). Early recognition and accurate diagnosis are vital, as cutaneous HCC metastasis generally indicates a poor prognosis and necessitates prompt management of both primary and metastatic lesions.^2^

## Conflicts of interest

None disclosed.
